# Genital Attacks in Hereditary Angioedema and Their Effects on Sexual Life

**DOI:** 10.3390/medicina60111777

**Published:** 2024-10-30

**Authors:** Asuman Camyar, Gokten Bulut, Melih Ozisik, Sevgi Altay, Ozlem Kuman Tuncel, Semiha Ozgul, Aytul Zerrin Sin, Nihal Mete Gokmen

**Affiliations:** 1 Department of Internal Medicine, Division of Immunology and Allergy, Faculty of Medicine, Ege University, Izmir 35100, Turkey; asuerden@yahoo.com (A.C.); goktenbulut58@gmail.com (G.B.); doktormelih@hotmail.com (M.O.); sevgialtay35@hotmail.com (S.A.); aytulsin@yahoo.com (A.Z.S.); 2 Department of Psychiatry, Faculty of Medicine, Ege University, Izmir 35100, Turkey; kumanozlem@yahoo.com; 3 Department of Biostatistics, Faculty of Medicine, Ege University, Izmir 35100, Turkey; semihaozgul@hotmail.com

**Keywords:** hereditary angioedema, genital attacks, attack triggers, sexual activity, sexual health

## Abstract

*Background and Objectives*: Hereditary angioedema (HAE) is characterized by unpredictable skin and mucosal angioedema attacks. We aimed to find the frequency of sexual-activity-triggered attacks (STAs) and understand how the sexual life of HAE with C1-inhibitor deficiency (HAE-C1INH) patients is affected. *Materials and Methods*: Adult HAE-C1INH patients were included in this cross-sectional study, which started in March 2020. Demographic information, marriage properties, gender-specific sexual life characteristics, and the HAE-specific histories of the patients were collected. The Hospital Anxiety and Depression Scale (HADS) and the Turkish version of the New Sexual Satisfaction Scale (NSSS) were applied to all participants. *Results*: Among 42 symptomatic HAE patients, 33 (78.57%) had genital attacks and 17 (42.5%) had STAs. Ten (58.8%) had genital pain, tenderness, or swelling, and five (29.4%) had isolated abdominal and groin pain. Eight (47.1%) patients with STAs experienced a HAE attack during their first time engaging in sexual intercourse. Anxiety/depression scales, NSSS scores, and distribution of other HAE attack localizations were similar in patients with and without STAs, and no gender differences were observed. Compared to the patients without STAs, the ratio of patients who stated that their sexual lives were negatively affected and that they lost their sexual desire was higher in patients with STAs. *Conclusions*: Genital or abdominal attacks triggered by sexual activity may be more common than thought. Sexual activity should also be questioned for evaluating attack triggers. There is a possibility of triggering an attack with the first and ongoing sexual intercourse, and patients should be informed to keep their attack treatment medications ready in advance.

## 1. Introduction

Hereditary angioedema (HAE) with C1-inhibitor deficiency (HAE-C1INH; Online Mendelian Inheritance in Man; OMIM #106100) is an autosomal dominant inherited rare disease characterized by unpredictable skin and mucosal angioedema.

There are two types of HAE-C1INH: type 1 and type 2. In type 1 HAE, patients have low C1 inhibitor (C1INH) antigenic levels, whereas, in type 2 HAE, patients have normal or elevated C1INH antigenic levels with defective C1-INH function [1].

C1 inhibitor (C1INH) is a glycoprotein that inhibits various serine proteases. With this inhibitor function, it regulates tissue contact activation, kallikrein, coagulation, and complement cascades [2]. Bradykinin is the responsible mediator of angioedema formation in HAE-C1INH patients [3]. Bradykinin is formed in the plasma from high-molecular-weight kininogen (HMWK) as a result of the catalysis of kallikrein and Factor XII. The binding of bradykinin to its receptor leads to the opening of endothelial cell junctions, resulting in fluid leakage into the tissues [4]. 

C1INH inhibits Factor XII, Factor XIIa and plasma kallikrein [2,5,6,7]. It has been shown that bradykinin levels increase and that FXII, plasma prekallikrein, and HMWK levels decrease during hereditary angioedema attacks [8].

Type 1 and type 2 HAE-C1INH share the same pathogenesis of angioedema, mutations in *SERPING1*, and dysfunctional C1INH, resulting in bradykinin overproduction. 

Patients experience recurrent angioedema attacks. The most involved skin areas are the extremities, face, genitals, and trunk/neck, with ratios of 93.8%, 75.6%, 62.7%, and 27.5%, respectively [9]. Angioedema attacks can affect the upper airways and cause laryngeal edema, carrying a risk of mortality if not treated effectively [10,11]. 

Genital edema can also occur in HAE. The buttocks, penis, perineum, pubis, scrotum, vagina, and vulva can be affected during angioedema attacks [12].

Stress and trauma are the best known attack triggers for HAE. Although sexual intercourse may include localized trauma, its role in developing HAE attacks has not been widely studied as a potential attack trigger. In a recent study, the ratio of patients who have genital attacks is reported as 81%, and, in 18% of them, sexual activity is found to be a triggering factor [12]. 

Sexual dysfunctions have been described in chronic diseases. Anxiety and depression also negatively affect sexual functions. Sexuality is a subject that is usually not discussed during patient follow-ups. Without being prompted by physicians, patients are reluctant to address sexual problems. There are no data in the literature on how the sexual lives of hereditary angioedema patients are affected [13]. 

Therefore, we aimed to find the frequency of HAE patients having attacks after sexual activity and to understand how the sexual life of patients with HAE is affected. 

We hypothesized that the actual frequency rate of genital attacks in HAE patients would be higher than reported and that sexual activity can initiate an attack and have negative impacts on patients’ sexual health. 

## 2. Methods

This cross-sectional study was approved by the Ethics Committee of Ege University (Approval Date: 19 February 2020—Approval Number: 19.02.2020-E.56477)

### 2.1. Patients

Adult HAE-C1INH patients under our clinic’s care who consented to participate were enrolled in the study conducted by the Department of Allergy and Clinical Immunology, Department of Internal Medicine, starting in March 2020. Written informed consent was obtained from all the participants after the details of the study were explained. HAE-C1INH diagnoses were made according to medical histories and low C1INH function values (50% or less than the standard reference levels). HAE-C1INH patients with low C1INH levels were categorized as HAE type 1 HAE (HAE-1), and HAE-C1INH patients with normal/high C1INH levels were classified as HAE type 2 HAE (HAE-2). All the patients included in the study from this center are supervised by a physician who has had personal/face-to-face follow-ups with these HAE-C1INH patients for years (NMG). 

### 2.2. Procedure

A case report questionnaire was developed to gather demographic information (gender, age, place of residence, education, marital status, marital types, employment status, income level, presence of chronic disease, presence of psychiatric disease, alcohol, and tobacco usage), gender-specific aspects of sexual life (birth control methods, masturbation causing genital attacks, having a sexual-activity-triggered attack (STA) upon first intercourse, exposure to sexual violence during intercourse, genital attacks having a negative effect on sexual life, number and type of births), and medical history related to HAE (HAE type, HAE onset age, HAE diagnosis age, attack localizations, having postpartum attacks).

For psychiatric evaluation, Turkish versions of the Hospital Anxiety and Depression Scale (HADS) and the New Sexual Satisfaction Scale (NSSS) were applied to all participants as self-report forms. The HADS was developed by Zigmond and Snaith [14]. The HADS has two subscales (anxiety (HADS-A) and depression (HADS-D)), and it is used for assessing the level, changes in severity, and the risk of anxiety and depression. The validity and reliability study of the HADS-TR in the Turkish language was reported by Aydemir et al., with cut-off points of 10 and 7, respectively, for the HADS-A and HADS-D [15]. 

Our study participants who scored above these cut-off values were accepted as having “anxiety disorder” or “depression”, respectively. The NSSS was developed by Štulhofera et al. (2010), and Tuğut performed its Turkish adaptation and validation study [16,17]. The NSSS was used to assess the sexual satisfaction of HAE patients. Higher scores define better sexual satisfaction. The NSSS has two subscales. The “ego-centered subscale” measures sexual satisfaction generated by personal experiences and sensations and the “partner-and sexual activity-centered subscale” measures sexual satisfaction derived from one’s partner’s sexual behaviors and reactions and the diversity or frequency of sexual activities.

Initially, an experienced nurse with specialized training in angioedema (SA) distributed self-reported questionnaires to the patients, and the patients filled out the case report questionnaires by themselves. Subsequently, within a week, all participants underwent a verbal interview conducted by their primary physician (NMG), during which the questionnaire was administered once more. After the oral interview, the answers given by the patients were evaluated as the final data. 

### 2.3. Statistical Analysis

Frequencies and percentages were given for categorical variables, and mean, standard deviation (SD), median, and range (minimum, maximum) values were given for numerical variables as descriptive statistics. A Mann–Whitney U test was used for two-group comparisons of numerical variables. The association between two categorical variables was analyzed using a Fisher’s exact test.

Statistical significance was assessed at *p* < 0.05, and all statistical analyses were performed using R software (R software, version 4.0.5, package: arsenal, R Foundation for Statistical Computing, Vienna, Austria).

## 3. Results

Forty-two symptomatic HAE patients were enrolled in this study. Among them, 21 patients (50%) were female, 36 (85.7%) patients had HAE-1, and six (14.7%) patients had HAE-2. The mean age at diagnosis was 29.4 ± 11.8, and the first attack age was 12.9 ± 9.7. Most participants (90.5%) lived in urban areas (Table 1). 

**Figure 1:**
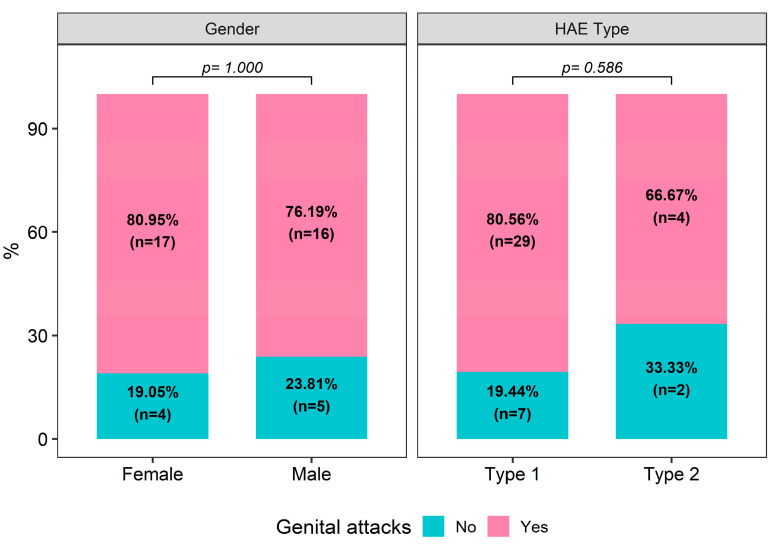
Distribution of genital attacks by gender and HAE type.

Among 42 patients, 33 (78.6%) of them had a genital attack history. Having genital attacks was not associated with gender or HAE type (*p* > 0.05 for both, Figure 1).

**Figure 2:**
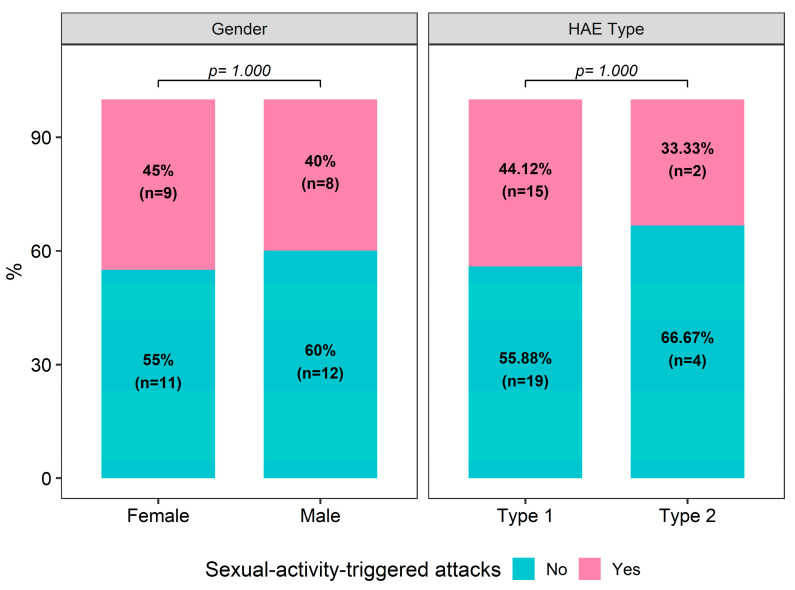
Distribution of sexual-activity-triggered attacks by gender and HAE type.

Seventeen patients (40.5%) had sexual-activity-triggered attacks (STAs). Genital pain, genital swelling, and tenderness were reported in ten of these patients (58.8%). Attacks in the abdomen and/or groin area without genital swelling were reported in five patients (29.4%) (Table 2). Having STAs was not associated with gender or HAE type (*p* = 1.000 for both, Figure 2).

**Figure 3:**
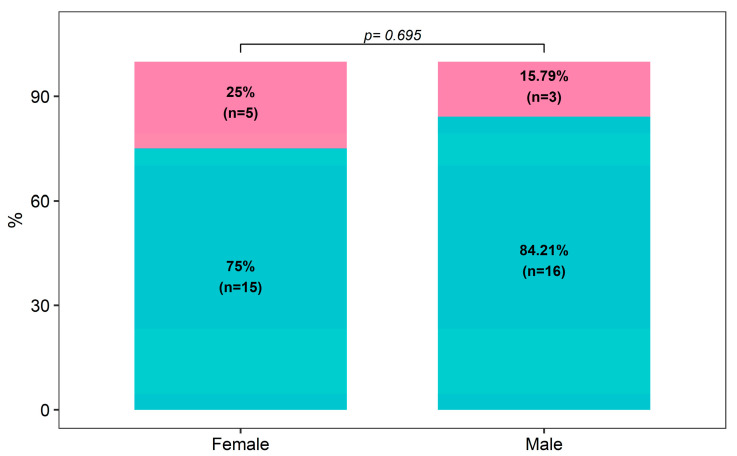
Distribution of sexual activity triggered attacks at first sexual intercourse by gender.

**Figure 4:**
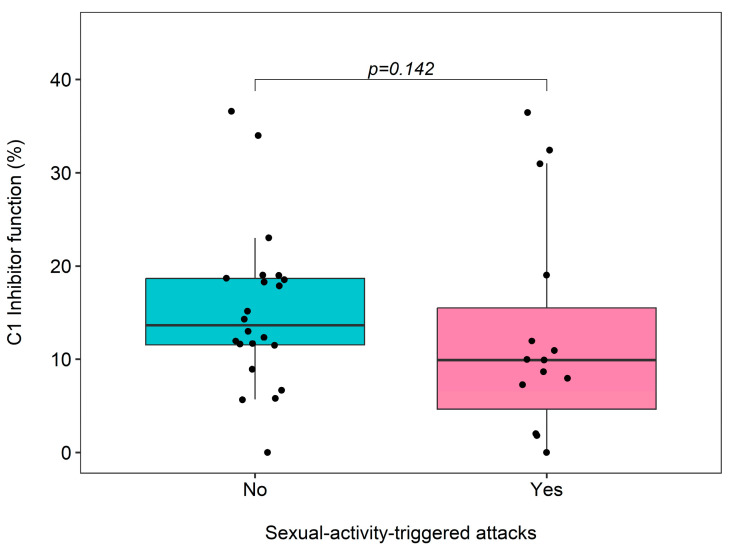
Comparison of C1 inhibitor function levels between patients experiencing sexual-activity-triggered attacks and those who do not. In the box plot, the bottom and top edges represent the first and third quartiles, respectively, while the horizontal line inside the box indicates the median. The lower and upper whiskers extend from the box up to 1.5 times the interquartile range (IQR). Points scattered around the box plot represent the individual measured values of C1 inhibitor function levels for each patient.

Eight of the patients (47.1%) who have STAs experienced an attack during or after their first engagement in sexual intercourse (Table 2), and the frequency of STAs did not differ between male and female patients (*p* = 0.695, Figure 3). There was no significant difference in C1 inhibitor function levels between those with STAs and those without (*p* = 0.142, Figure 4).

In the comparison of patients with and without STAs, no difference was observed in terms of marital status, education, place of residence, income level, type of marriage, and distribution of other HAE attack localizations (all *p* values *p* > 0.05). 

Sixteen patients (94.1%) who experienced STAs stated ‘Yes’ to the question “Is your sexual life negatively affected due to genital attacks?”. In those without STAs, this rate was 43.5% (*p* = 0.001). 

The scale scores of the patients who said their sexual life was negatively affected by genital attacks and the scores of those who said they were not affected are shown in Table 3. According to demographic properties (gender, place of residence, education, marital status, marital types, employment status, income level), no differences were observed in terms of reporting that their sexual life was negatively affected (all *p* values *p* > 0.05). 

The NSSS and HADS scores of the patients did not differ according to STA status and having an STA after first intercourse. Demographic properties (gender, place of residence, education, marital status, marital types, employment status, income level, presence of psychiatric disease, alcohol or tobacco use) were also found to have no effect on the NSSS and HADS scores (all *p* values *p* > 0.05). 

All of the patients with STAs reported a loss of sexual desire and avoidance from their partner. The frequency of loss of sexual desire among patients without STAs is 66.7% (*p* = 0.014). 

None of our patients who gave birth vaginally (*n* = 10) received short-term prophylaxis (STP), and postpartum attacks were reported in one (10%) of them. Two out of three patients (66.7%) who received prophylaxis before a cesarean section developed an attack. One out of four patients (25%) who did not receive prophylaxis before cesarean section had a birth-giving-related attack. 

The viral serologic work-up of sexually transmitted viruses (HBV, HCV, and HIV) was negative in all 42 patients. 

## 4. Discussion

In this study, we investigated the effects of genital attacks and sexual-activity-triggered attacks (STAs) in hereditary angioedema. Physical trauma and stress are the most frequently reported triggers in HAE, with one study reporting stress as a trigger in 60% of patients and trauma as a trigger in 47% of patients [18]

In our previous study, the prevalence of HAE-C1INH patients who have genital attacks was 85% [19]. The genital attack rate was reported to be 62.7% in the study of Bork 2006 [9]. In a recent study by Mormile et al. 2022, the genital attack ratio was 81% in HAE-C1INH patients [12]. Our study, similar to this recent study, showed that the rate of patients experiencing genital attacks was 78.6%. 

The effect of sexual activity on HAE attacks has been neglected in past studies. Sexual activity was reported as a triggering factor in 18% of the patients with genital attacks [12]. 

In our study, the rate of patients having a HAE attack after sexual intercourse is 42.5% (17 out of 42), and almost half of the patients (47.1%) who have STAs stated that they had an attack after their first engagement in sexual intercourse. For this reason, STP may be performed before first sexual intercourse, or medications that patients can use during an acute attack should be prepared in advance. Attacks caused by masturbation were found to be at a lower rate than attacks caused by sexual intercourse (19% vs. 42.7%). The sexual activity patterns of the patients were not questioned. There might be differences between masturbation and other sexual activities in terms of physical trauma. We recommend educating all patients about this subject and reassuring them to have a healthy sexual life. 

The abdomen is the most common attack location in regard to HAE [20,21,22]. Abdominal pain is generally accepted as a type of attack that begins spontaneously. Patients may experience an abdominal attack as abdominal pain, a feeling of bloating in the abdomen, or groin pain. Abdominal pain during the attacks was reported as severe in 52% of patients [18]. In our study, isolated abdominal or groin pain, without the involvement of another site, was reported in 29.4% as an STA. 

In HAE patients presenting with abdominal attacks, sexual activity should also be questioned when evaluating the attack trigger. 

Depression and anxiety were previously reported in HAE patients [23]. In one study, the elevated anxiety and depression scores were 21% and 23% in the general population based on the HADS [24]. In our study, the anxiety disorder risk was 15%, the depression risk was 32.5%, and, in the patients having genital attacks or STAs, the HADS scores were not found to be higher (*p* > 0.05). It was previously published that the attack severity and the attack frequency did not have any influence on the HADS scores [18]. We similarly observed that having genital attacks or STAs did not impose an additional burden in terms of depression and anxiety risks in our patients. 

There is also a social burden on the patients and their caregivers. Family life and overall everyday productivity are negatively affected by the disease [21,25]. This may further disrupt intimate relationships between spouses. 

The NSSS scores of our patients who had genital attacks or STAs were not lower. However, a significant number of patients directly responded that genital attacks negatively affected their sexual lives. The negative feelings about the sexual life of the patients might be attributed to the disease itself, not to the attack sites. A second possible reason is that the patients may have responded more sincerely to a question about a topic that is still considered taboo when directly asked by a physician who has been following them for many years (NMG). 

Although the risk of developing a HAE attack is high, the incidence of HAE attacks during vaginal delivery is relatively low and considered to be safer than with cesarean sections [26,27]. It is recommended that STP is performed for patients who are planning to have a cesarean section, who have had a vacuum or forceps delivery, who have frequent attacks in the third trimester, or who have trauma-induced genital attacks [1,28,29,30]. Therefore, the use of C1-inhibitor concentrate for STP is not routinely recommended during vaginal delivery. The rate of attacks following vaginal birth in this study is 10%. Due to the low sample size in this rare disease, a clear risk could not be stated. We recommend STP before vaginal delivery for women with frequent HAE attacks. Since STP is not always effective, close observation for up to three days postpartum is mandatory for all patients who are giving birth. 

It has been reported that androgen use may affect the sexual life of patients [31]. Our study was not designed as a prospective cohort. No standard statistical data could be obtained to assess the effects of the androgens, as our patients have used androgens as short-term and long-term prophylaxis at different times of their lives. Due to the current health policies, other new agents used in long-term prophylaxis (e.g., lanedelumab or berotralstat) have not yet been included in the treatment of our patients. 

## 5. Conclusions

In conclusion, genital or abdominal attacks triggered by sexual activity may be more common than previously thought. Sexual activity should be evaluated as a potential triggering factor. This issue, which is challenging to discuss openly, should be discussed with a trusted physician, and patients should be assured that they will receive the medical care they need. There is a possibility of triggering an attack during a patient’s first sexual intercourse, and patients should be informed to keep their attack treatment medications ready in advance.

## Figures and Tables

**Table 1:** Sociodemographic and medical characteristics of the sample (*n* = 42).

	HAE-Type I (N = 36)	HAE-Type II (N = 6)	Total (N = 42)	*p* Value
Gender				0.663
Female	19 (52.8%)	2 (33.3%)	21 (50.0%)	
Male	17 (47.2%)	4 (66.7%)	21 (50.0%)	
Age (years), mean ± SD	41.5 ± 14.8	36.3 ± 5.1	40.8 ± 13.9	0.323
HAE onset age (years), mean ± SD	13.5 ± 10.2	9.3 ± 5.0	12.9 ± 9.7	0.296
HAE diagnosis age (years), median (min, max)	30.0 (10.0, 58.0)	25.0 (15.0, 33.0)	29.0 (10.0, 58.0)	0.109
Place of residence				0.474
Village	1 (2.8%)	1 (16.7%)	2 (4.8%)	
Town	2 (5.6%)	0 (0.0%)	2 (4.8%)	
City	33 (91.7%)	5 (83.3%)	38 (90.5%)	
Last graduated school				0.627
Primary school	11 (30.6%)	1 (16.7%)	12 (28.6%)	
Middle School	11 (30.6%)	1 (16.7%)	12 (28.6%)	
University	14 (38.9%)	4 (66.7%)	18 (42.9%)	
Marital status				0.567
married	24 (66.7%)	6 (100.0%)	30 (71.4%)	
never married	9 (25.0%)	0 (0.0%)	9 (21.4%)	
divorced	2 (5.6%)	0 (0.0%)	2 (4.8%)	
widow	1 (2.8%)	0 (0.0%)	1 (2.4%)	
Marriage types, (N-miss)	9	0	9	0.379
love marriage	11 (40.7%)	1 (16.7%)	12 (36.4%)	
arranged marriage	16 (59.3%)	5 (83.3%)	21 (63.6%)	
The age at marriage (years), mean ± SD	24.3 ± 5.8	25.3 ± 2.9	24.5 ± 5.4	0.181
Family types (N-miss)				1.000
nuclear	34 (94.4%)	6 (100.0%)	40 (95.2%)	
extended	2 (5.6%)	0 (0.0%)	2 (4.8%)	
Number of children, median (min, max) (N-miss)	12 2 (1, 3)	0 1 (1, 2)	12 2 (1, 3)	0.032
Employment status, (N-miss)				0.472
not working	6 (16.7%)	1 (16.7%)	7 (16.7%)	
working	18 (50.0%)	5 (83.3%)	23 (54.8%)	
retired	10 (27.8%)	0 (0.0%)	10 (23.8%)	
student	2 (5.6%)	0 (0.0%)	2 (4.8%)	
Income level, (N-miss)				0.032
low	29 (80.6%)	2 (33.3%)	31 (73.8%)	
medium	7 (19.4%)	4 (66.7%)	11 (26.2%)	
Alcohol use	6 (16.7%)	1 (16.7%)	7 (16.7%)	1.000
Smoking	4 (11.1%)	1 (16.7%)	5 (11.9%)	
Alcohol use + Smoking	2 (5.6%)	0 (0%)	2 (4.8%)	
None	24 (66.7%)	4 (66.7%)	28 (66.7%)	
Chronic disease	7 (19.4%)	0 (0.0%)	7 (16.7%)	0.567
Psychiatric disease	3 (8.3%)	0 (0.0%)	3 (7.1%)	1.000

HAE: hereditary angioedema; SD: standard deviation; N-miss: number of missing values. Unless otherwise stated in the table, data are presented as *n* (%).

**Table 2:** HAE-specific sexual life characteristics of patients.

	HAE-Type I (N = 36)	HAE-Type II (N = 6)	Total N = 42	*p* Value
Use of birth control (N-miss)	3	0	3	1.000
Yes	19 (57.6%)	4 (66.7%)	23 (59.0%)	
No	14 (42.4%)	2 (33.3%)	16 (41.0%)	
Birth control method (N-miss)	17	2	19	0.605
Condom	9 (47.4%)	1 (25.0%)	10 (43.5%)	
Oral contraceptive pills	1 (5.3%)	0 (0.0%)	1 (4.3%)	
Withdrawal	3 (15.8%)	2 (50.0%)	5 (21.7%)	
Intrauterine device	5 (26.3%)	1 (25.0%)	6 (26.1%)	
Tubal ligation	1 (5.3%)	0 (0.0%)	1 (4.3%)	
Subjected to violence during sexual intercourse due to the genital attack (N-miss)	3	1	4	1.000
Yes	4 (12.1%)	0 (0.0%)	4 (10.5%)	
No	29 (87.9%)	5 (100.0%)	34 (89.5%)	
Sexual-activity-triggered attacks (N-miss)	2	0	2	1.000
Yes	15 (44.1%)	2 (33.3%)	17 (42.5%)	
No	19 (55.9%)	4 (66.7%)	23 (57.5%)	
Patient complaints and findings of sexual-activity-triggered attacks (N-miss)	4	0	4	0.338
No attack	19 (59.4%)	4 (66.7%)	23 (60.5%)	
Genital Swelling	1 (3.1%)	1 (16.7%)	2 (5.3%)	
Abdominal Pain	1 (3.1%)	1 (16.7%)	2 (5.3%)	
Groin Pain	3 (9.4%)	0 (0.0%)	3 (7.9%)	
Genital Pain And Tenderness	5 (15.6%)	0 (0.0%)	5 (13.1%)	
All Of Them	3 (9.4%)	0 (0.0%)	3 (7.9%)	
Attacks developed with the first sexual intercourse (N-miss)	3	0	3	1.000
Yes	7 (21.2%)	1 (16.7%)	8 (20.5%)	
No	26 (78.8%)	5 (83.3%)	31 (79.5%)	
Attacks with masturbation				1.000
Yes	7 (19.4%)	1 (16.7%)	8 (19.0%)	
No	29 (80.6%)	5 (83.3%)	34 (81.0%)	
Whether genital attacks negatively affect sexual life (N-miss)	2	0	2	0.279
Yes	29 (85.3%)	4 (66.7%)	33 (82.5%)	
No	5 (14.7%)	2 (33.3%)	7 (17.5%)	
Impact of genital attack on sexual life (N-miss)	3	1	4	0.661
No effect	4 (12.1%)	1 (20.0%)	5 (13.2%)	
Loss of sexual desire	1 (3.0%)	0 (0.0%)	1 (2.6%)	
Avoidance from the partner	1 (3.0%)	0 (0.0%)	1 (2.6%)	
Loss of sexual desire and avoidance from the partner	27 (81.8%)	4 (80.0%)	31 (81.6%)	

N-miss: number of missing values. Unless otherwise stated in the table, data are presented as *n* (%).

**Table 3:** The scale scores of those who answered yes and those who answered no to the question “Is your sexual life negatively affected due to genital attacks?”.

	Yes (N = 33)	No (N = 7)	Total (N = 40)	*p* Value
NSSS total score				0.546
N-Miss	2	0	2	
Median (Range)	73.0 (20.0, 99.0)	71.0 (43.0, 75.0)	73.0 (20.0, 99.0)	
Ego-centered NSSS score				0.727
N-Miss	3	0	3	
Median (Range)	34.5 (10.0, 50.0)	35.0 (32.0, 40.0)	35.0 (10.0, 50.0)	
Partner-centered NSSS score				0.545
N-Miss	2	0	2	
Median (Range)	36.0 (10.0, 49.0)	36.0 (11.0, 38.0)	36.0 (10.0, 49.0)	
HADS-anxiety score				0.720
Median (Range)	8.0 (2.0, 17.0)	7.0 (2.0, 12.0)	7.5 (2.0, 17.0)	
HADS-depression score				0.567
Median (Range)	5.0 (1.0, 15.0)	5.0 (1.0, 9.0)	5.0 (1.0, 15.0)	
Anxiety disorder risk				1.000
Yes	5 (15.2%)	1 (14.3%)	6 (15.0%)	
No	28 (84.8%)	6 (85.7%)	34 (85.0%)	
Depression risk				1.000
Yes	11 (33.3%)	2 (28.6%)	13 (32.5%)	
No	22 (66.7%)	5 (71.4%)	27 (67.5%)	

NSSS: New Sexual Satisfaction Scale, HADS: Hospital Anxiety and Depression Scale, N-Miss: number of missing values. Unless otherwise stated in the table, data are presented as *n* (%).

## Data Availability

Data are available on request due to restrictions. The data presented in this study are available on request from the corresponding author due to patient confidentiality.

## Abbreviations

HAE	Hereditary Angioedema
C1INH	C1 Inhibitor
HAE-C1INH	Hereditary Angioedema with C1-inhibitor deficiency
HAE-1	Hereditary Angioedema Type 1
HAE-2	Hereditary Angioedema Type 2
HADS	The Hospital Anxiety and Depression Scale
NSSS	New Sexual Satisfaction Scale
STA	Sexual-Activity-Triggered Attacks
STP	Short-Term Prohylaxis
